# Comparative clinical response, safety, and institutional drug use efficiency of intravenous azithromycin versus erythromycin in pediatric *Mycoplasma pneumoniae* pneumonia: a real-world evidence study

**DOI:** 10.3389/fcimb.2026.1782840

**Published:** 2026-02-13

**Authors:** Jiayu Deng, Yifei Li, Changxin Liu, Xiaoyu Qu, Yanqing Song

**Affiliations:** 1 Department of Pharmacy, The First Hospital of Jilin University, Changchun, China; 2 Department of Pediatrics, The First Hospital of Jilin University, Changchun, China; 3 School of Pharmaceutical Sciences, Jilin University, Changchun, China

**Keywords:** azithromycin, erythromycin lactobionate, intravenous macrolides, medication efficiency, *Mycoplasma pneumoniae* pneumonia, propensity score matching, real-world evidence, sparse-event safety

## Abstract

**Objective:**

This study aims to compare real-world clinical response, safety, and institutional medication efficiency of intravenous (IV) azithromycin (AZI) versus erythromycin lactobionate (ERY) in hospitalized children with *Mycoplasma pneumoniae* pneumonia (MPP).

**Methods:**

A retrospective cohort of 1,049 children with PCR- or serology-confirmed MPP was assembled (AZI: *n* = 672; ERY: *n* = 377). Propensity scores were estimated using prespecified baseline confounders (sex, age, severity phenotype, concomitant antibacterial agents, antiviral co-treatment). A 1:1 nearest-neighbor propensity score matching (PSM) without replacement cohort was built (364 matched pairs per arm). The primary endpoint was a three-level composite ordinal outcome (cure, improvement, ineffective) hierarchically assigned at 72 ± 12 h after IV macrolide initiation, without assuming that missing domains imply success. Two sensitivity cohorts tested missingness assumptions. Secondary endpoints included LOS, macrolide duration, and corticosteroid escalation, interpreted as adaptive process nodes. Sparse safety used bias-reduced likelihood inference. Institutional drug efficiency was evaluated by decomposing macrolide costs into dispensed, consumed, and wastage-related avoidable cost signals.

**Results:**

Before matching, ordinal response distributions differed modestly (*P* = 0.040). After PSM, composite ordinal outcomes were similar (paired ordinal *P* = 0.599), with comparable cure rates (33.8% vs. 32.7%). A treatment × age interaction signal was observed (*P*_interaction=0.008). In smaller strata (<80 per arm), ORs for a higher ordinal grade with ERY vs. AZI were 0.75 (<8 years; *P* = 0.096) and 2.19 (≥8 years; *P* = 0.029). In the adjusted full cohort, ERY showed higher odds of mostly mild adverse events (adjusted OR 6.52, *P* = 0.006), driven by skin reactions (adjusted OR 17.90, *P* = 0.021) with wide CIs from sparse precision. Institutional macrolide costs were substantially higher with ERY (both *P* < 0.001). Duration was longer with ERY (*P* < 0.001), while LOS and escalation rates were similar post-match.

**Conclusions:**

IV AZI and IV ERY showed comparable overall clinical response in hospitalized pediatric MPP. The age interaction is a response-heterogeneity signal requiring confirmation, not causal proof of efficacy reversal. ERY carried higher odds of mostly mild adverse events, longer duration, and greater institutional macrolide cost burden. These results support future work on resistance-informed, sequence-aware, and child-appropriate formulation stewardship to improve interpretability, safety precision, and institutional antibiotic sustainability.

## 1 Introduction

*Mycoplasma pneumoniae* (*M. pneumoniae*) pneumonia (MPP) is one of the most common causes of pediatric community-acquired pneumonia (CAP) requiring hospitalization in China and contributes to substantial seasonal inpatient surges, particularly among school-aged children. Intravenous (IV) macrolides remain the empirical backbone of inpatient therapy, yet the high prevalence of macrolide-resistant *M. pneumoniae* and antimicrobial-stewardship-linked institutional efficiency pressures have created uncertainty surrounding early drug selection, safety monitoring, and medication use sustainability (Jiang et al., 2023; Chen et al., 2024; Han et al., 2025).

Clinically, macrolide-unresponsive MPP (MUMPP) is identified when children fail to show symptoms or radiologic improvement after 72 h of standard IV macrolide therapy. Although tetracyclines and fluoroquinolones are recognized escalation options for refractory or severe MPP, their use is restricted in younger children due to short-term safety concerns (e.g., dental effects or musculoskeletal toxicity signals), making macrolides the default initial IV therapy for most hospitalized children (Yang, 2019; Ahn et al., 2021; Wang et al., 2024).

Among IV macrolide agents used at high frequency in pediatric inpatient care, azithromycin (AZI) and erythromycin lactobionate (ERY) are the most commonly administered therapies for atypical pneumonias. While prior trials and cohorts suggest broadly comparable short-term effectiveness, direct real-world evidence (RWE) contrasting these agents across multidomain ordinal clinical response, rare safety events, and decomposed patient-level economic efficiency, including avoidable medication wastage under China’s hospital payment reforms [Diagnosis-Related Groups (DRG)/Diagnosis-Intervention Packet (DIP)], remains limited, particularly when inefficiency-related costs accrue to institutions rather than patients (Berger et al., 2009; Austin, 2011; Smale et al., 2021).

To address these evidence gaps, we conducted a single-center, retrospective, hypothesis-driven, propensity score-matched real-world cohort study comparing IV AZI versus IV ERY in hospitalized children with MPP. We prespecified hypotheses of (1) overall comparable multidomain ordinal composite clinical response after confounding control, (2) age-dependent heterogeneity in treatment process or response architecture (<8 vs. ≥8 years) without assuming deterministic drug causality, and (3) differential short-term safety and institutional medication use efficiency, including pharmacy-linked avoidable cost separation, to inform pediatric antimicrobial stewardship and pharmacy systems decision-making (Austin, 2010; Chatton et al., 2020).

## 2 Methods

### 2.1 Study design and data source

This was a single-center, retrospective, real-world comparative-effectiveness cohort study in hospitalized children with MPP at the Lequn Branch, The First Hospital of Jilin University, a tertiary academic medical center. Structured patient-level data were retrieved from the hospital electronic medical record (EMR) and health information system (HIS) and abstracted using a standardized, hypothesis-driven structured case report form (CRF). Extracted variables included demographics (sex, age), clinical severity phenotype (mild vs. severe), concomitant antibacterial and antiviral therapy, multidomain clinical response trajectories (symptom, laboratory, and radiographic), IV macrolide treatment duration, corticosteroid escalation, sparse safety events (hematologic, gastrointestinal, skin), macrolide drug costs (dispensed and consumed), and hospital length of stay (LOS).

The analysis plan, endpoint hierarchy, model choices, and treatment × age interaction testing were finalized prior to outcome modeling and effect estimation, consistent with current credibility standards for prespecified RWE designs (Berger et al., 2009; Austin, 2011; Chatton et al., 2020). All effectiveness and safety outcomes were attributed to the index IV macrolide exposure using an intention-to-treat (ITT)-like causal contrast narrative without assuming deterministic age-dependent drug efficacy.

Ethical approval was granted by the Ethics Committee of The First Hospital of Jilin University (approval no. 2025-016). As a retrospective design using fully anonymized administrative and clinical data, the requirement for informed consent was waived.

### 2.2 Study population and exposure definition

The source population included children hospitalized at the Lequn Branch, The First Hospital of Jilin University, with PCR- or serology-supported MPP from January 1, 2023 to December 31, 2024. MPP was identified by (1) a positive *M. pneumoniae* PCR or IV macrolide-triggering serology (IgM positivity or a fourfold IgG rise when available) and (2) radiologist- or clinician-documented pneumonia features (fever, cough, lobar/segmental consolidation, or interstitial infiltrates) without another pathogen fully explaining the syndrome.

The inclusion criteria required children to be <18 years, to be initiated on IV AZI or IV ERY for ≥72 consecutive hours as the index macrolide, and to have ≥1 evaluable clinical domain at 72 ± 12 h after initiation (symptom, laboratory, or radiographic trajectory). This preserves real-world macrolide selection contrast at cohort entry and supports paired causal-contrast inference in the matched cohort (Berger et al., 2009; Austin, 2011; 2014).

Exclusion criteria were applied to remove children whose early trajectories could be dominated by non-index drug or non-pneumonia conditions, including hospital LOS <72 h, macrolide exposure <72 h, within-course switching between AZI and ERY, ICU admission for non-infectious indications before macrolide initiation, or major comorbid conditions that could independently distort clinical or safety trajectories (congenital heart disease, acute or chronic hepatic or renal insufficiency, neurologic disorders impairing baseline respiratory assessment, bacterial sepsis, or bloodstream infection). Children who discontinued the index macrolide, were discharged, or required treatment modification before completing 72 h of therapy were excluded to preserve a consistent exposure contrast and avoid attribution ambiguity in early clinical trajectories.

Baseline variables were defined as those recorded at hospital admission or within the first 24 h of hospitalization, prior to or immediately following initiation of the index IV macrolide. The index exposure time (*t* = 0) was defined as the start of the first eligible IV macrolide administered for ≥72 consecutive hours. Outcome assessment was conducted at approximately 72 ± 12 h after index macrolide initiation, reflecting routine clinical reassessment timing in hospitalized pediatric pneumonia. This temporal framework aligns cohort entry, exposure definition, and outcome assessment within a unified real-world care timeline.

Exposure definition (index macrolide) was assigned at the patient level by the first eligible IV macrolide administered for ≥72 consecutive hours after admission, emulating an ITT-like real-world framework where all downstream outcomes were attributed to the index exposure group without assuming deterministic pharmacologic age effects (Berger et al., 2009; Austin, 2011; 2014). Children were categorized into (1) IV AZI group and (2) IV ERY group. This attribution strategy preserves empirical drug selection contrasts, avoids age stratum rematching bias, and ensures that interactions are interpreted as response or prescribing-context heterogeneity signals rather than causal efficacy reversals.

### 2.3 Propensity score estimation and matching

Propensity scores (PS) for index IV macrolide exposure (AZI vs. ERY) were estimated at the patient level using a multivariable logistic regression model, where the dependent variable was the index exposure assignment. Covariates were finalized prior to outcome modeling and prespecified based on established confounding control guidance for pediatric antimicrobial comparative effectiveness research, including sex, age (continuous, years), baseline clinical severity phenotype (mild vs. severe MPP), concomitant use of non-macrolide antibacterial agents, and concomitant antiviral therapy. The PS model development followed recommended standards for design, estimation, and transparent reporting in RWE cohorts (Berger et al., 2009; Austin, 2010; 2011; 2014).

Covariates for PS estimation were selected *a priori* to reflect baseline patient characteristics and early clinical severity available at cohort entry while avoiding inclusion of post-exposure or pathway-mediated variables. Laboratory biomarkers, radiographic evolution, oxygen escalation, and corticosteroid initiation were not included in the propensity model because they may reflect early treatment response or downstream clinician decision-making rather than baseline severity. This covariate selection strategy was intended to preserve causal interpretability of the initial IV macrolide selection contrast rather than adjust away real-world prescribing behavior.

A 1:1 nearest-neighbor matching without replacement design was applied on the logit-transformed PS, using a caliper width equal to 0.2 × SD of the logit(PS) to avoid poor matches and reduce residual bias while preserving precision, consistent with validated RWE matching performance standards (Austin, 2011; 2014). Because this rule is SD-based, the absolute caliper value varied across analytic settings depending on the underlying distribution of the logit-transformed propensity score, and the realized caliper applied in each analysis is reported where relevant.

No further rematching was performed within age strata after propensity score matching (PSM), ensuring that age contributed to confounding control as a continuous baseline confounder and effect-modification models were entered only once as a treatment × age_group interaction term, preserving the empirical causal contrast of initial IV macrolide choice and avoiding age stratum rematching bias.

Covariate balance after matching was evaluated using standardized mean differences (SMD), with SMD <0.10 prespecified as the threshold for adequate balance diagnostics following established appraisal guidance for matched cohort comparability (Mamdani et al., 2005; Berger et al., 2009; Austin, 2011).

### 2.4 Outcomes

#### 2.4.1 Primary outcome

The primary outcome was overall clinical efficacy at approximately 72 ± 12 h after initiation of the index IV macrolide, captured as a three-level composite ordinal endpoint: cure, improvement, or ineffective. This multidomain endpoint was designed to reflect real-world pediatric pneumonia care logic, integrating symptom resolution, laboratory response, and radiographic evolution, as recommended in pediatric pneumonia research and practice guidelines that emphasize combined clinical, biomarker, and imaging assessments for treatment response evaluation (Cho et al., 2019; Butpech and Tovichien, 2025).

Symptom trajectory was coded from clinician documentation and vital sign records. A code of 0 (non-response) was assigned to children with persistent fever (temperature ≥37.8°C) or ongoing respiratory symptoms (e.g., cough, dyspnea, oxygen dependency) without clear improvement by 72 h. A code of 1 (response-emergent) was assigned when a child achieved sustained defervescence (temperature <37.5°C for ≥24 h) and respiratory symptom improvement was documented, consistent with the symptomatic response criteria used in observational MPP cohorts (Cho et al., 2019).

Laboratory trajectory was coded from routinely available inflammatory biomarkers, focusing on C-reactive protein (CRP) and D-dimer levels, both of which have been shown to correlate with disease severity and treatment response in pediatric lower respiratory infections. A code of 0 indicated no improvement or worsening biomarker trends, 1 indicated a downward trend without normalization, and 2 indicated normalization of both biomarkers (CRP <10 mg/L and D-dimer <0.5 mg/L). Missing biomarker data were coded as NA (Zheng et al., 2021).

Radiographic trajectory was coded based on structured chest imaging reports. A code of 0 denoted consolidation or infiltrates that did not improve or progress, 1 described partial absorption or non-resolved improvement, and 2 described radiologist-reported lesion resolution, substantial absorption, or explicit remission/cure descriptions. Radiographic findings are well-established markers of disease progression and correlate with clinical severity in pediatric pneumonia, including MPP (Cho et al., 2019).

Laboratory (CRP, D-dimer) and radiographic domains were incorporated as supportive components of the composite ordinal outcome rather than independent efficacy endpoints. Symptom trajectory was prioritized in outcome classification, while laboratory and imaging findings functioned as confirmatory or veto domains. Delayed normalization of biomarkers or radiographic findings did not automatically imply treatment failure, and missing laboratory or imaging data never implied cure.

The composite ordinal classification was assigned using predefined hierarchical rules. A child was classified as ineffective if the symptom code was 0 or if both laboratory and radiographic domains were available and both were coded 0 (dual non-response). Cure required symptom improvement (symptom = 1) plus at least one companion domain normalized (laboratory = 2 or radiographic = 2), but no available domain could be coded 0 (veto rule against cure under any observed non-response). Children with symptom improvement who did not meet the cure criteria were classified as improvement; laboratory or radiographic missingness (NA) did not automatically imply cure and was permitted only within the improvement category.

#### 2.4.2 Sensitivity analyses for the primary outcome

To evaluate the robustness of the three-level composite ordinal endpoint under alternative missing-data assumptions, two prespecified sensitivity analyses were performed. Sensitivity analyses were defined before model fitting to emulate RWE credibility standards, where outcome missingness is addressed through complementary cohort restrictions rather than imputation that assumes clinical success by default (Berger et al., 2009; Austin, 2011).

Sensitivity analysis 1 (complete-case cohort, 1:1 PSM with replacement) was restricted to children with both laboratory and radiographic outcome domains observed (non-missing) at 72 ± 12 h after index macrolide initiation (lab ≠ NA and imaging ≠ NA). Within this restricted cohort, PS were re-estimated, followed by 1:1 nearest-neighbor PSM with replacement, a validated approach to preserve sample size and avoid poor matches in smaller cohorts without inducing additional age-stratum bias. The composite ordinal endpoint was then re-calculated using identical cure/improvement/ineffective rules, ensuring comparability of the causal contrast with the main analysis (Mamdani et al., 2005; Chatton et al., 2020).

Sensitivity analysis 2 (excluding double-missing cases, 1:1 PSM without replacement) removed children with both laboratory and radiographic domains simultaneously missing (lab = NA and imaging = NA) before PS estimation. PS were re-estimated, followed by 1:1 nearest-neighbor matching without replacement using the same logit-PS caliper logic as the main analysis. The composite ordinal endpoint was then re-constructed under unchanged hierarchical rules, avoiding any assumption that missing data represent cure or clinical success (Austin, 2011; Puhr et al., 2017).

For ordinal inference in both sensitivity cohorts, paired Wilcoxon signed-rank tests and Bowker’s symmetry tests were applied, with robust variance clustered by matched pair ID to account for within-pair correlation. In all analyses, missing domains never implied cure, preserving an adaptive, phenotype-driven real-world response assessment narrative rather than pathway-agnostic causal claims (Berger et al., 2009; Austin, 2011; Olson et al., 2020).

#### 2.4.3 Secondary outcomes

Secondary endpoints covered three complementary domains: effectiveness, short-term safety, and treatment-process variables. Effectiveness endpoints included hospital LOS (days) and index IV macrolide treatment duration (days or hours). Treatment-process heterogeneity endpoints included treatment escalation, defined as systemic corticosteroid co-prescription initiated during the index IV macrolide course (1 = yes, 0 = no), a real-world adaptive prescribing node commonly used in pediatric pneumonia cohorts to modulate inflammatory phenotype rather than to replace antimicrobial class by default (Lipshaw et al., 2021; Chen et al., 2024).

Safety endpoints were coded at the patient level as 1 (occurred) or 0 (not occurred), including (1) hematologic adverse events (e.g., platelet decline, leukopenia, or coagulopathy flagged by clinicians), (2) gastrointestinal (GI) adverse events (e.g., nausea, vomiting, diarrhea, or abdominal intolerance documented after IV macrolide exposure), and (3) skin reactions (e.g., rash, pruritus, or infusion-related erythema). Any adverse event was defined as any of the above safety domains coded as 1. Given sparse safety events in pediatric MPP RWE cohorts, bias-reduced inference for adjusted safety ORs used Firth-penalized logistic regression for all safety endpoints to stabilize estimates and avoid separation-driven exaggeration (Austin, 2011; Puhr et al., 2017).

Secondary outcomes were compared before and after PSM using paired or correlation-aware inference. For matched cohorts, Wilcoxon signed-rank tests were applied for skewed continuous endpoints, McNemar’s test for binary endpoints, and pair-clustered regression for multivariable uncertainty estimation, consistent with RWE matched-cohort appraisal standards (Mamdani et al., 2005; Berger et al., 2009; Austin, 2011; Chatton et al., 2020).

Electrocardiographic abnormalities (including QT interval prolongation), liver function test (LFT) elevations, and severe hypersensitivity reactions were not included as predefined safety endpoints. In routine pediatric MPP care at our institution, ECG monitoring and serial LFT measurements are not systematically performed in otherwise stable children receiving short-course IV macrolides, resulting in incomplete and non-standardized data capture. In addition, repeated day-by-day blood sampling for liver enzyme monitoring is generally avoided in pediatric inpatients to minimize procedural burden and discomfort, further limiting the feasibility of longitudinal LFT assessment. As a result, these outcomes could not be reliably analyzed within a comparative framework. GI adverse events in this study were limited to clinically documented symptoms (e.g., nausea, vomiting, diarrhea) and did not include biochemical liver enzyme abnormalities.

To evaluate age-dependent heterogeneity without inducing age-stratum rematching bias, interaction tests (treatment × age group, <8 vs. ≥8 years) were performed in the original PSM-matched cohort without further rematching, using cluster-robust standard errors by matched pair ID and reported as *P*_interaction. This preserves the causal-contrast narrative that interaction signals reflect real-world prescribing or recovery-architecture heterogeneity rather than deterministic pharmacologic efficacy modification by age (Torres et al., 2015; Yang, 2019; Olson et al., 2020).

#### 2.4.4 Economic outcomes and drug efficiency

Economic drug efficiency was assessed by decomposing IV macrolide costs into (1) dispensed cost, derived from HIS billing records at the dispensing-unit level, and (2) consumed cost, recalculated based on the actual number of vials administered to each child during the index macrolide course. Notably, IV AZI/ERY in our institution uses fixed-vial billing rather than milligram-granular, weight-based micro-dose costing, a common architecture in pediatric hospital pharmacy systems that can generate narrow patient-level dispensed cost distributions (Toerper et al., 2014; Fan et al., 2024).

To aid in the interpretation of drug wastage, institutional dosing and preparation practices are briefly described. IV AZI and ERY were prescribed using weight-based pediatric dosing regimens, with dose rounding constrained by fixed commercial vial sizes available in the hospital pharmacy. IV AZI was supplied in single-use vials that frequently exceeded per-dose requirements in younger or lower-weight children, whereas ERY was dispensed in smaller or more flexibly divisible vial configurations. Drug preparation followed routine pharmacy reconstitution workflows, and partially used vials could not be reassigned to other patients for sterility and safety reasons. As a result, unused drug remaining after dose preparation was recorded as wastage. These practices reflect standard inpatient pharmacy operations rather than discretionary clinician behavior.

The difference (dispensed − consumed) was reported as a wastage-related avoidable cost signal, reflecting formulation–utilization misfit, vial size to pediatric weight mismatch, or batch preparation workflow constraints. Similar decomposition strategies have been validated to quantify institutional medication inefficiency independent of clinical drug effect, aligning with sustainable medication supply and antimicrobial stewardship evaluation frameworks (Berger et al., 2009; Austin, 2011; Smale et al., 2021).

Consistent with RWE principles, wastage cost was interpreted strictly as a health system process inefficiency indicator, not a measure of clinical treatment failure, intrinsic drug inferiority, or a causal estimand of antimicrobial effectiveness. Pediatric pharmacy literature underscores that medication waste is structurally driven by packaging–utilization architecture rather than drug performance itself, especially under reform-era hospital payment systems where avoidable costs accrue to institutions (Toerper et al., 2014; Smale et al., 2021; Fan et al., 2024).

### 2.5 Age subgroup analysis and interaction testing

Age was prespecified as a potential effect modifier at the design stage, grounded in clinical practice where tetracyclines become permissible escalation options in children ≥8 years, potentially shaping distinct treatment and recovery pathways in macrolide-managed pediatric MPP (Ahn et al., 2021; Wang et al., 2024). Prespecifying effect modifiers before modeling is recommended for credibility in RWE comparative-effectiveness research (Berger et al., 2009; Austin, 2011).

Two age strata were defined: <8 years and ≥8 years. Ordinal treatment-age heterogeneity was evaluated using a cumulative-logit proportional odds ordinal logistic regression model, the canonical modeling framework for ordered clinical outcomes, recommended for ranked therapeutic response inference (McCullagh, 1980; Agresti, 2010). This approach is widely adopted in pediatric infectious disease RWE studies to evaluate ordered response constructs while preserving matched correlation (Jiang et al., 2023; Meyer Sauteur et al., 2024).

Effect modification was tested by including a treatment × age_group interaction term using cluster-robust standard errors at the matched pair level to account for within-pair correlation, consistent with recommended matched-inference practices in RWE antibiotic comparative-effectiveness research (Berger et al., 2009; Austin, 2011; Chatton et al., 2020). Interaction *P*-values were reported using Wald tests, and effect sizes were expressed as exponentiated odds ratios (ORs) with 95% confidence intervals, consistent with standard reporting for ordinal treatment effects in matched cohorts (Chatton et al., 2020).

Secondary binary endpoints were analyzed using pair-clustered logistic regression to preserve matched correlation. Sparse safety endpoints were modeled using Firth-penalized logistic regression, a recommended bias-reduced approach for rare-event antibiotic safety inference under quasi- or complete separation (Heinze and Schemper, 2002; Puhr et al., 2017).

Skewed positive continuous endpoints, including LOS and drug costs, were modeled using a log-linked Gamma generalized linear model (Gamma GLMs), the canonical framework for right-skewed healthcare and treatment-process cost outcomes, without assuming equal variance across dispensing arms (Malehi et al., 2015; Deb and Norton, 2018).

### 2.6 Statistical analysis

Baseline comparability before matching was evaluated using chi-square (*χ*²) tests or Fisher’s exact tests for categorical variables, and *t* tests or Mann–Whitney *U* tests for continuous variables based on distributional assumptions. This approach is consistent with recommended observational cohort reporting for pediatric pneumonia RWE cohorts (Austin, 2011; Malehi et al., 2015).

Inference after PSM applied paired or correlation-aware methods to account for within-pair dependency and to generate valid variance estimates in matched RWE cohorts. McNemar’s test was used for binary endpoints, paired Wilcoxon signed-rank tests for skewed continuous endpoints, and Bowker’s symmetry tests for the three-level composite ordinal endpoint; preserving matched-pair correlation without assuming missing domains imply clinical success (Berger et al., 2009; Austin, 2011). Hospital LOS and macrolide treatment duration exhibited right-skewed distributions and were therefore summarized using medians and interquartile ranges and analyzed using non-parametric tests or log-linked Gamma GLMs as appropriate.

Ordered clinical response effects were modeled using cumulative-logit proportional odds ordinal logistic regression, the canonical regression framework for ranked clinical effectiveness endpoints. The proportional odds (parallel slopes) assumption underlying the cumulative-logit model was assessed, and no meaningful violation was detected. This model is the accepted standard for three-level or *n*-level ordered efficacy outcomes in infectious disease RWE cohorts (McCullagh, 1980; Austin, 2010).

All sparse safety endpoints (hematologic, gastrointestinal, skin reactions) were analyzed using Firth-penalized logistic regression, a bias-reduced likelihood method recommended for rare-event antibiotic safety inference in cohorts where separation or quasi-separation may occur. This unified modeling strategy avoids continuity-correction inconsistency and stabilizes effect estimates under sparse counts (Heinze and Schemper, 2002; Puhr et al., 2017).

Institutional cost and duration outcomes, which were strictly positive and heavily right-skewed, were modeled using Gamma GLMs with log link, the standard parametric framework for skewed hospital pharmacy cost data and antibiotic treatment-process variables (Malehi et al., 2015; Smale et al., 2021).

Population-marginal estimands (marginal means and risk differences) were derived using g-computation with individual-level counterfactual prediction and cohort averaging, a recommended approach for estimating population-average contrasts in matched or unmatched RWE antibiotic cohorts (Austin, 2011; Chatton et al., 2020).

All hypothesis tests were two-sided, with *P <*0.05 indicating statistical significance. Post-matching covariate balance used SMD <0.10 as the adequacy threshold, consistent with best-practice diagnostics for pediatric antimicrobial comparative-effectiveness matched cohorts (Berger et al., 2009; Austin, 2011).

## 3 Results

### 3.1 Study population and baseline characteristics

**Figure 1:**
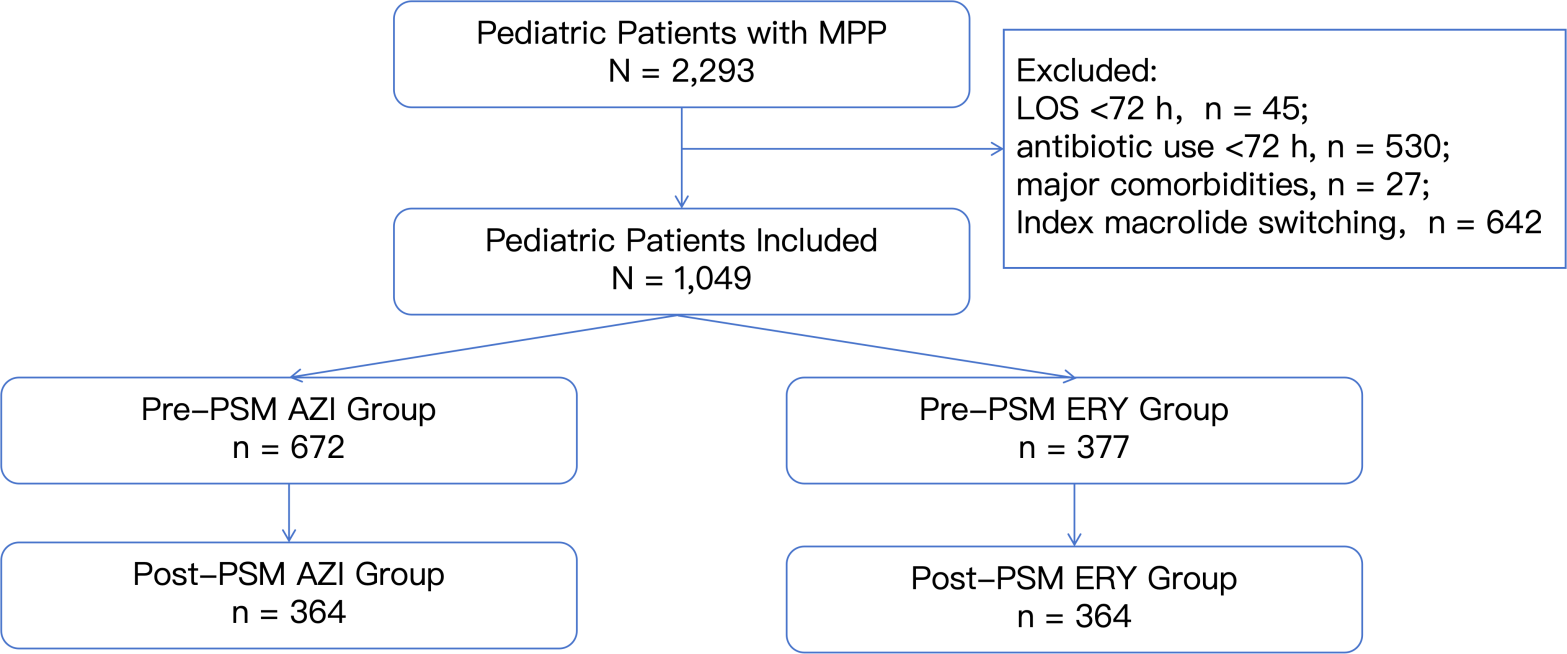
Cohort screening and selection flowchart.

A total of 1,049 hospitalized children diagnosed with MPP were included in the analysis, comprising 672 patients treated with AZI and 377 treated with ERY (**Figure 1**). A 1:1 nearest-neighbor PSM without replacement was subsequently performed, adjusting for sex, age, clinical severity phenotype, concomitant antibacterial therapy, and antiviral co-treatment, resulting in 364 matched pairs per group (**Table 1**).

**Table 1:** Baseline before and after PSM.

Variable	Pre-PSM			Post-PSM		
	AZI (*n* = 672)	ERY (*n* = 377)	SMD	AZI (*n* = 364)	ERY (*n* = 364)	SMD
Male, *n* (%)	350 (52.1)	189 (50.1)	0.039	190 (52.2%)	184 (50.5%)	0.033
Age, years (median, IQR)	4.00 (2.92, 6.00)	5.00 (2.83, 7.00)	0.211	4.00 (2.90, 7.00)	4.50 (2.83, 7.00)	0.062
Severe phenotype, *n* (%)	108 (16.1%)	94 (24.9%)	0.220	77 (21.2%)	83 (22.8%)	0.040
Concomitant antibacterial therapy, *n* (%)	575 (85.6%)	319 (84.6%)	0.027	314 (86.3%)	308 (84.6%)	0.047
Concomitant antiviral therapy, *n* (%)	447 (66.5%)	275 (72.9%)	0.140	267 (73.4%)	262 (72.0%)	0.031

PSM, propensity score matching; SMD, standardized mean difference; IQR, interquartile range.

Before matching, baseline characteristics were generally comparable, although slight imbalances were noted in age distribution (median 4.0 vs. 5.0 years, SMD = 0.211) and severe phenotype prevalence (16.1% vs. 24.9%, SMD = 0.220). After matching, covariate balance substantially improved across all variables, with SMD values <0.10, including male proportion (52.2% vs. 50.5%, SMD = 0.033), median age (4.0 vs. 4.5 years, SMD = 0.062), severe phenotype (21.2% vs. 22.8%, SMD = 0.040), concomitant antibacterial therapy (86.3% vs. 84.6%, SMD = 0.047), and antiviral co-treatment (73.4% vs. 72.0%, SMD = 0.031), indicating adequate post-PSM balance for comparative effectiveness assessment.

### 3.2 Primary outcome

Before matching, AZI-treated children (*n* = 672) achieved cure in 255 cases (37.9%), improvement in 409 (60.9%), and ineffective response in eight (1.2%). In the ERY cohort (*n* = 377), cure occurred in 122 patients (32.4%), improvement in 245 (65.0%), and ineffective response in 10 (2.7%). Although the overall efficacy distribution showed a marginal difference between groups (*χ*² test, *P* = 0.056), an ordinal comparison detected a modest ordered shift in clinical response (Mann–Whitney *U* test, *P* = 0.040), supporting between-group heterogeneity in the ranked composite efficacy outcome (**Table 2**).

**Table 2:** Primary outcome before and after PSM.

Outcome	Pre-PSM		Post-PSM	
	AZI (*n* = 672)	ERY (*n* = 377)	AZI (*n* = 364)	ERY (*n* = 364)
Cure, *n* (%)	255 (37.9%)	122 (32.4%)	123 (33.8%)	119 (32.7%)
Improvement, *n* (%)	409 (60.9%)	245 (65.0%)	235 (64.6%)	236 (64.8%)
Ineffective, *n* (%)	8 (1.2%)	10 (2.7%)	6 (1.6%)	9 (2.5%)
Panel comparison	Unmatched ordinal test		Paired matched ordinal test	
Ordinal test *P*-value	Mann–Whitney U, *P* = 0.040^a^		Wilcoxon signed-rank, *P* = 0.599	
Distribution test *P*-value	*χ*² test, *P* = 0.056		Bowker’s test, *P* = 0.819	

Matching method: 1:1 nearest-neighbor matching without replacement with a caliper width defined as 0.2 × SD of the logit-transformed propensity score. The realized absolute caliper value in the primary matched cohort was 0.071, restricting matched pairs to an absolute PS difference ≤0.071. Values are presented as *n* (%) or *n*. After matching, *P*-values correspond to paired tests. Clinical efficacy was evaluated using a three-level composite ordinal endpoint.

PSM, propensity score matching.

a *P*-value <0.05 is considered to indicate a significant difference.

After 1:1 nearest-neighbor PSM without replacement, 364 matched pairs were generated. In the matched cohort, the distribution of the three-level composite clinical efficacy outcome was well balanced between treatment arms (AZI: cure, 123 [33.8%]; improvement, 235 [64.6%]; ineffective, six [1.6%]; ERY: cure, 119 [32.7%]; improvement, 236 [64.8%]; ineffective, nine [2.5%]). Paired ordinal tests showed no statistically significant difference in ordered composite efficacy between ERY and AZI within the matched sample (Wilcoxon signed-rank test, *P* = 0.599; Bowker’s symmetry test, *P* = 0.819; **Table 2**).

The proportional odds assumption was satisfied for the composite ordinal clinical efficacy outcome, supporting the use of a cumulative-logit modeling framework.

### 3.3 Sensitivity analyses of the primary outcome

To evaluate the robustness of the primary outcome against different missing-data handling strategies, two complementary sensitivity analyses were performed.

Sensitivity analysis 1 (complete-case, 1:1 PSM with replacement): A complete-case cohort was constructed by including only children with both laboratory and chest imaging outcome components available, yielding 167 AZI-treated and 135 ERY-treated patients before matching. Substantial baseline imbalance was observed, particularly in age (SMD = 0.492) and severe phenotype prevalence (SMD = 0.342). After 1:1 nearest-neighbor PSM with replacement using a caliper width defined as 0.2 × SD of the logit-transformed propensity score (realized absolute caliper value = 0.175, restricting the maximum absolute PS difference to ≤0.175), 133 matched pairs per group were generated with substantially improved, though not fully adequate, post-matching covariate balance, as reflected by a residual SMD for age of 0.212 (**Supplementary Table S1**). No statistically significant difference in the ordered composite efficacy endpoint was detected (paired Wilcoxon signed-rank test, *P* = 0.581), and the paired categorical composition showed no distributional asymmetry (Bowker’s test for paired nominal data, *P* = 0.236), indicating that the complete-case inference was robust to stricter outcome-component availability requirements (**Supplementary Table S2**).

Sensitivity analysis 2 (excluding double-missing cases, 1:1 PSM without replacement, caliper = 0.086): To further test endpoint stability, children with missing both laboratory and imaging components were excluded prior to matching. This produced 577 AZI-treated and 302 ERY-treated cases before matching. After 1:1 nearest-neighbor PSM without replacement (caliper = 0.086), 286 matched pairs per group were retained. As in sensitivity 1, baseline covariate balance was well achieved after matching (**Supplementary Table S3**). No significant difference in ordered clinical efficacy was found (paired Wilcoxon, *P* = 0.810), and no asymmetry was detected in paired categorical composition (Bowker’s test, *P* = 0.319) (**Supplementary Table S4**).

Across both sensitivity analyses, the direction, magnitude, and statistical inference of the primary outcome remained consistent with the main analysis, supporting the robustness of the conclusions that no significant difference exists between AZI and ERY on the three-level composite clinical efficacy endpoint after adequate confounding control. Sensitivity 1 additionally demonstrated that control reuse under replacement matching did not materially alter ordinal efficacy inference, while sensitivity 2 confirmed that excluding double-missing outcome components did not introduce hidden ordinal effects. Collectively, these results reinforce endpoint stability and the reliability of the primary findings.

### 3.4 Age subgroup analysis and interaction test

To evaluate potential effect modification by age, children were stratified into <8 years and ≥8 years, based on clinical considerations that tetracyclines are commonly reserved as alternative therapy for patients aged ≥8 years, which may introduce divergent treatment pathways. In the PSM-matched cohort, no re-matching was performed within age strata to preserve the original matched sample. A three-level ordinal logistic regression model (cumulative logit) was applied Instead, including treatment group, age group, and a treatment × age group interaction term. For inference, robust standard errors clustered by matched pairs were used to account for within-pair correlation, and the Wald test of the interaction coefficient was reported as *P*_interaction (**Table 3**).

**Table 3:** Age-stratified composite clinical efficacy and treatment × age interaction test in the PSM-matched cohort.

Outcomes	AZI, *n*	ERY, *n*	OR (95% CI)	*P*-value
<8 years	*n* = 285	*n* = 287	0.75 (0.54, 1.05)	0.096
Cure, *n* (%)	105 (36.8%)	89 (31.0%)		
Improvement, *n* (%)	178 (62.5%)	192 (66.9%)		
Ineffective, *n* (%)	2 (0.7%)	6 (2.1%)		
≥8 years	*n* = 79	*n* = 77	2.19 (1.08, 4.42)	0.029^a^
Cure, *n* (%)	18 (22.8%)	30 (39.0%)		
Improvement, *n* (%)	57 (72.2%)	44 (57.1%)		
Ineffective, *n* (%)	4 (5.1%)	3 (3.9%)		
*P*_interaction (treatment × age group)			0.008	

ORs were estimated via cumulative logit ordinal logistic regression. Robust SEs clustered by pair_id. Interaction was tested using Wald test of treatment × age_group.

OR, odds ratio; CI, confidence interval.

a *P*-value <0.05 is considered to indicate a significant difference.

In children aged <8 years (AZI: *n* = 285; ERY: *n* = 287), outcome frequencies were cure 105 (36.8%) vs. 89 (31.0%), improvement 178 (62.5%) vs. 192 (66.9%), and ineffective 2 (0.7%) vs. 6 (2.1%). No statistically significant difference was detected between ERY and AZI on the composite ordered endpoint in the matched cohort (ordinal logistic regression, OR = 0.75, 95% CI 0.54–1.05, *P* = 0.096), indicating comparable ordered clinical response within this age stratum.

In children aged ≥8 years (AZI: *n* = 79; ERY: *n* = 77), outcomes were cure 18 (22.8%) vs. 30 (39.0%), improvement 57 (72.2%) vs. 44 (57.1%), and ineffective 4 (5.1%) vs. 3 (3.9%). In this subgroup, ERY was associated with significantly higher-ordered composite efficacy (OR = 2.19, 95% CI 1.08–4.42, *P* = 0.029).

The treatment × age group interaction term was statistically significant (*P*_interaction = 0.008), demonstrating that the relative ordered efficacy of ERY vs. AZI differed between age strata, supporting the presence of age-dependent treatment effect heterogeneity (effect modification).

### 3.5 Secondary outcomes and age-stratified effect modification

In the PSM-matched pediatric cohort, median LOS was comparable between treatment arms (AZI: 6.83 [5.76, 8.89] days vs. ERY: 6.98 [5.78, 8.04] days; *P* = 0.475). The proportion of children receiving treatment escalation with systemic corticosteroids also showed no significant difference (186 [51.1%] vs. 177 [48.6%]; *P* = 0.536). However, macrolide treatment duration was markedly longer with ERY (6.00 [5.00, 7.00] days vs. 4.00 [4.00, 4.00] days; *P* < 0.001) (**Table 4**).

**Table 4:** Secondary outcomes before and after PSM.

Variable	Pre-PSM			Post-PSM		
	AZI (*n* = 672)	ERY (*n* = 377)	*P*-value	AZI (*n* = 364)	ERY (*n* = 364)	*P*-value
LOS, days, (median, IQR)	6.84 (5.77, 8.01)	6.98 (5.79, 8.06)	0.132	6.91 (5.78, 8.89)	6.98 (5.78, 8.04)	0.475
Macrolide treatment duration, days, (median, IQR)	4.00 (4.00, 4.00)	6.00 (5.00, 7.00)	<0.001^a^	4.00 (4.00, 4.00)	6.00 (5.00, 7.00)	<0.001^a^
Treatment escalation (systemic corticosteroid use), *n* (%)	320 (47.6%)	186 (49.3%)	0.638	186 (51.1%)	177 (48.6%)	0.536

PSM, propensity score matching; LOS, length of stay.

a *P*-value <0.05 is considered to indicate a significant difference.

To further evaluate age-dependent effect modification, we analyzed three prespecified strata (<8 vs. ≥8 years) using regression models preserving the original matched sample and clustering inference by pair_id.

For LOS, log-transformed OLS regression showed no significant difference in either stratum (<8 years ratio = 0.99 [0.94–1.04], *P* = 0.666; ≥8 years ratio = 0.99 [0.90–1.09], *P* = 0.837), with no detectable treatment × age interaction (*P*_interaction *>*0.999) (**Supplementary Table S5**).

For treatment duration, ERY was associated with significantly longer therapy in both age strata (<8 years ratio = 1.44 [1.37–1.52], *P* < 0.001; ≥8 years ratio = 1.60 [1.43–1.79], *P* < 0.001). The treatment × age interaction suggested stronger separation in ≥8 years, but did not reach statistical significance (*P*_interaction = 0.096) (**Supplementary Table S6**).

For corticosteroid escalation, logistic regression confirmed no significant between-group difference (<8 years OR = 0.89 [0.66–1.21], *P* = 0.471; ≥8 years OR = 0.95 [0.50–1.82], *P* = 0.885) and no significant treatment × age interaction (*P*_interaction = 0.858). A paired exact binomial McNemar test for symmetry within matched pairs similarly supported no stratum-specific difference (*P* = 0.654 and 0.761 for <8 and ≥8 years) (**Supplementary Table S7**).

These findings were together directionally consistent with the main analysis, supporting robust inference for secondary endpoints despite outcome component restriction, matching design variation, and control reuse.

### 3.6 Safety outcomes

The overall incidence of safety events was low in both groups (**Table 5**). Unadjusted comparisons were performed using Fisher’s exact test due to sparse event counts, showing a higher rate of any adverse event in the ERY group compared with the AZI group (7/377 [1.9%] vs. 2/672 [0.3%], *P* = 0.013), and skin reactions occurred exclusively in the ERY arm (4/377 [1.1%] vs. 0/672 [0.0%], *P* = 0.017).

**Table 5:** Safety outcomes in the full, unmatched cohort: Comparison between AZI and ERY.

Safety endpoint	AZI (*n* = 672)	ERY (*n* = 377)	RD (ERY – AZI), %	OR (95% CI)	*P*-value
Adverse event, *n* (%)	2 (0.3%)	7 (1.9%)	1.6	5.43 (1.29, 22.85)	0.013^a^
Hematologic event, *n* (%)	1 (0.1%)	0 (0.0%)	-0.1	0.59 (0.02, 14.59)	>0.999
Gastrointestinal event, *n* (%)	1 (0.1%)	3 (0.8%)	0.6	4.18 (0.62, 28.45)	0.135
Skin event, *n* (%)	0 (0.0%)	4 (1.1%)	1.1	16.20 (0.87, 301.82)	0.017^a^

ORs and 95% CIs were estimated with a continuity correction (Haldane–Anscombe, +0.5 to all cells) to address zero-event separation in some safety endpoints. *P*-values were computed using Fisher’s exact test.

RD, risk difference; OR, odds ratio; CI, confidence interval.

a *P*-value <0.05 is considered to indicate a significant difference.

Given sparse events and evidence of quasi-separation, Firth-penalized logistic regression was applied for adjusted inference, confirming that ERY remained associated with higher odds of any adverse event (adjusted OR = 6.52, 95% CI 1.73–24.53, *P* = 0.006) and skin reactions (adjusted OR = 17.90, 95% CI 1.56–205.67, *P* = 0.021) (**Supplementary Table S8**).

### 3.7 Economic outcomes

Economic evaluation was performed in the full pediatric cohort using complete drug cost records from the hospital information system. Prior to adjustment, ERY incurred a substantially higher dispensed macrolide drug cost compared with AZI (median 695.80 [497.00–1,192.80] CNY vs. 80.84 [80.84–80.84] CNY; Mann–Whitney U test, *P* < 0.001) (**Table 6**). After multivariable adjustment using Gamma GLMs with log link and HC1-robust standard errors, including sex, standardized age (z-score), clinical severity phenotype, concomitant antibacterial therapy, and concomitant antiviral therapy, the cost burden remained significantly higher with ERY (adjusted cost ratio 9.84 [9.39–10.31], *P* < 0.001). G-computation-derived marginal means also supported this difference (82.53 CNY vs. 812.23 CNY for AZI vs. ERY).

**Table 6:** Economic outcomes in the full cohort: Macrolide drug costs and wastage comparison between AZI and ERY.

Economic outcomes	AZI (*n* = 672)	ERY (*n* = 377)	Unadjusted *P*-value	Adjusted ratio (ERY/AZI) (95% CI)	Adjusted *P*-value	Adjusted marginal mean AZI	Adjusted marginal mean ERY
Dispensed (CNY), mean ± SD/median, IQR	80.36 ± 17.89/80.84 (80.84, 80.84)	850.31 ± 431.30/695.80 (497.00, 1192.80)	<0.001^a^	9.84 (9.39, 10.31)	<0.001^a^	82.53	812.23
Actual use (CNY), mean ± SD/median, IQR	32.33 ± 18.54/26.68 (21.02, 36.38)	676.15 ± 390.56/596.40 (397.60, 894.60)	<0.001^a^	18.76 (17.91, 19.65)	<0.001^a^	33.95	636.97
Avoidable cost (wastage) (CNY), mean ± SD/median, IQR	48.03 ± 18.92/51.74 (40.42, 59.82)	174.15 ± 158.12/143.14 (31.81, 278.32)	<0.001^a^			46.71	188.22

Unadjusted *P*-values were computed using Mann–Whitney *U* test. Adjusted ratios for dispensed and actual macrolide costs were estimated using Gamma GLMs with log link, adjusting for sex, standardized age (z-score), clinical severity, concomitant antibacterial use, and concomitant antiviral use, with robust (HC1) standard errors. Adjusted marginal means were obtained by g-computation (predicting each individual’s expected cost under treat = 0 and treat = 1, then averaging). Wastage costs were modeled using a two-part approach (logistic regression for any wastage >0 CNY and gamma regression for positive wastage amount), and marginal means were derived from the combined model estimates. Ratio >1 indicates a higher or longer cost burden with ERY versus AZI. aP-value <0.05 is considered to indicate a significant difference.

For actual macrolide expenditure (based on consumption rather than dispensing), ERY also showed a significantly higher cost (median 596.40 [397.60–894.60] CNY vs. 26.68 [21.02–36.38] CNY; *P* < 0.001). After covariate adjustment with the same Gamma-GLM framework, the cost ratio (ERY/AZI) was 18.76 [17.91–19.65] (*P* < 0.001), with corresponding marginal means of 33.95 CNY vs. 636.97 CNY.

To quantify avoidable cost due to wastage (dispensed cost − actual consumption), a two-part model was prespecified given the high frequency of zero-wastage cases and positive right-skew among non-zero values. In the unmatched cohort, AZI wastage was more common (646/672 [96.1%]) than ERY (290/377 [76.9%]), with a significant unadjusted difference (*P* < 0.001) (**Supplementary Table S9**). After adjustment for the probability of any wastage (>0 CNY), ERY was associated with a significantly lower likelihood of any wastage (adjusted OR 0.13 [0.09–0.21], *P* < 0.001). However, among children who experienced wastage, ERY produced markedly higher avoidable costs (adjusted ratio 4.78 [4.42–5.18], *P* < 0.001). Two-part G-computation suggested a mean avoidable cost per treated child of 46.71 CNY vs. 188.22 CNY.

At the cohort level, the total dispensed macrolide cost was 374,566.12 CNY, of which 97,932.81 CNY (26.1%) represented avoidable wastage (**Supplementary Table S10**). Within treatment arms, wastage proportion was 59.8% for AZI and 20.5% for ERY. These data highlight a theoretical opportunity for further cost reduction through child-appropriate formulations or unit-dose packaging while maintaining efficacy, aligning with antimicrobial stewardship goals.

## 4 Discussion

This study provides RWE that IV macrolide treatment for pediatric MPP occurs within multimodal inpatient care pathways, where measurable drug-class signals may be smaller than the influence of phenotype-aware prescribing and adaptive treatment processes. In China, macrolides remain the empirical first-line choice for hospitalized children with MPP despite rising resistance pressures and limited randomized evidence directly comparing IV macrolide formulations (Meyer Sauteur, 2024; Wang et al., 2024).

The modest ordinal efficacy signal observed before matching and its disappearance after confounder balancing support that initial IV macrolide selection is coupled to baseline severity and clinician pathway triage, rather than separable intrinsic drug efficacy. Such coupling between antibiotic initiation choice and disease phenotype is well recognized in observational infectious-disease cohorts and does not imply deterministic drug inferiority (Mamdani et al., 2005; Austin, 2011). Accordingly, the absence of a statistically significant difference after confounder balancing reflects a lack of detectable difference under routine clinical conditions rather than constituting formal evidence of equivalence.

Treatment duration behaved as an adaptive process endpoint, where IV ERY was more commonly extended in children with slower early recovery. High-quality pediatric CAP trials confirm that longer macrolide exposure does not imply higher efficacy, as shorter 5-day strategies are non-inferior to 10-day courses among clinically improving children, reinforcing that duration extension reflects compensatory clinician response to delayed recovery rather than a stronger antimicrobial effect (Pernica et al., 2021).

Although a treatment × age interaction signal reached statistical significance, its interpretation requires caution. In real-world pediatric pneumonia research, significant interaction terms in matched cohorts without age-stratum rematching are commonly interpreted as response or prescribing-architecture heterogeneity signals, not causal proof of deterministic age-dependent drug efficacy (Olson et al., 2020; Lipshaw et al., 2021).

From a short-term safety perspective, ERY showed higher odds of any adverse event and mild skin reactions, but absolute event counts remained small and did not imply severe toxicity. In sparse-data antibiotic safety cohorts, the uniform use of Firth-penalized logistic models is recommended to avoid exaggeration of safety ORs due to separation (Austin, 2011; Puhr et al., 2017).

The economic component demonstrates that medication inefficiency is structurally distinct from clinical drug performance. Pediatric pharmacy literature confirms that avoidable costs often arise from vial-size mismatch to pediatric weight and batch dispensing architecture, producing institutional waste signals even when drug efficacy is broadly comparable (Toerper et al., 2014; Fan et al., 2024). Differences in wastage were driven primarily by vial-size mismatch and preparation constraints under weight-based pediatric dosing rather than by prescribing inefficiency or drug efficacy differences.

Finally, wastage behaved in a probability–magnitude dichotomy, a known pattern in sustainable medication supply studies, where waste probability and per-unit cost magnitude may diverge depending on vial architecture, batch preparation, and formulation-fit constraints. Importantly, this wastage signal reflects health-system inefficiency rather than antimicrobial failure and has clear stewardship relevance for pediatric hospitals operating under DRG/DIP payment reforms where avoidable costs accrue to institutions rather than patients (Smale et al., 2021).

## 5 Limitations and future directions

The limitations of this study stem from restricted observability of clinical decision pathways and treatment sequencing rather than cohort size alone. First, the retrospective dataset cannot fully recover latent clinical reasoning, real-time antibiotic extension triggers, or the precise timing of adaptive treatment nodes, including vial-level dosing frequency, batch preparation constraints, and corticosteroid initiation heuristics. These unobserved factors may influence treatment duration, exposure extension, and intensification decisions. Null findings in observational comparative-effectiveness studies should be interpreted as absence of detectable difference under the study design rather than as formal evidence of therapeutic equivalence.

Although the propensity model incorporated key baseline severity proxies, residual confounding from unmeasured clinical indicators (e.g., subtle respiratory distress, clinician gestalt) cannot be excluded. In addition, while no strong temporal prescribing shifts or service-level clustering patterns were observed during the study period, the single-center design limits formal disentanglement of time-period effects or unit-specific prescribing preferences. These factors warrant further evaluation in multicenter or target-trial–emulated studies with richer severity and provider-level data. In addition, in the complete-case sensitivity analysis, post-matching balance for age improved substantially but remained above the prespecified SMD <0.10 threshold, indicating residual imbalance that should be considered when interpreting these sensitivity findings.

Second, by design, children who improved sufficiently to discontinue therapy or be discharged before 72 h, as well as those who deteriorated rapidly and required early treatment modification, were excluded. This may preferentially retain clinically stable inpatient trajectories and limit inference for very early responders or non-responders. Accordingly, the findings should be interpreted as reflecting treatment effects within typical hospitalized pediatric MPP care pathways rather than the full spectrum of early disease evolution.

Third, although age was included in baseline confounding control, the cohort was not rematched within age strata after PSM, meaning that interaction signals may still reflect clinician pathway channeling, phenotype-aware treatment extension, and institutional drug utilization architecture. This introduces residual subgroup confounding that cannot be fully deconvoluted from drug-class effects.

Fourth, the safety cohort contained sparse adverse events, limiting inferential precision. While bias-reduced likelihood methods such as Firth-penalized logistic regression can stabilize coefficient estimation under separation risk, the very low absolute incidence of macrolide-associated adverse events inherently constrains statistical power and results in wide confidence intervals. Moreover, in pediatric inpatient practice, frequent serial blood testing for liver enzymes is often avoided to reduce procedural burden, which limits the availability of longitudinal biochemical safety data. Accordingly, safety findings should be interpreted as descriptive signals rather than definitive comparative toxicity estimates.

Finally, avoidable wastage cost estimates reflect institution-specific pharmacy workflow, packaging-utilization fit, and seasonal workload constraints. These signals should be interpreted as health-system stewardship inefficiency indicators, not intrinsic drug attributes, causal efficacy estimands, or deterministic signals of drug failure.

Future research should prioritize resistance-aware and sequence-capturing target-trial frameworks for pediatric MPP, explicitly modeling process nodes such as dosing frequency, treatment timing, corticosteroid initiation timing, and clinician-driven pathway selection. Prospective or multicenter pediatric MPP cohorts are needed to (1) validate how early clinical trajectories triage children into extended-exposure macrolide pathways, (2) characterize corticosteroid timing architecture and macrolide dose-frequency–response tradeoffs as process-level causal variables, and (3) evaluate pediatric unit-of-use formulation and packaging fit as institutional stewardship interventions using decomposed cost and pharmacy time-cost burden capture.

## 6 Conclusion

From a RWE perspective, this study demonstrates that IV macrolide treatment effects in pediatric MPP are tightly coupled to baseline clinical phenotype selection and adaptive care-process decisions, making short-term effectiveness signals difficult to isolate from the broader treatment pathway architecture. The most robust insights generated by this analysis relate to pathway-driven effect dilution, compensatory exposure extension, and formulation–utilization misfit, rather than deterministic evidence of intrinsic drug superiority or inferiority. These findings highlight that pediatric antimicrobial RWE studies should prioritize explicit modeling of clinical decision nodes, treatment timing, and formulation fit to better separate pharmacologic signals from institutional medication-use efficiency constraints. Future work should evolve toward resistance-aware, sequence-capturing designs and child-appropriate formulation stewardship research, aiming to optimize both clinical interpretability of treatment pathways and health-system efficiency without overstating causal drug effects.

## Data Availability

The raw data supporting the conclusions of this article are available from the corresponding author upon reasonable request. Requests to access these datasets should be directed to JD, dengjiayu@jlu.edu.cn.
