# Supplementary material for: Comparative clinical response, safety, and institutional drug use efficiency of intravenous azithromycin versus erythromycin in pediatric Mycoplasma pneumoniae pneumonia: a real-world evidence study
Source: Front Cell Infect Microbiol. 2026 Feb 13;16:1782840. doi: 10.3389/fcimb.2026.1782840 (PMC12945838; doi:10.3389/fcimb.2026.1782840)
Supplement: Supplementary file 1 [file Table1.docx]

Supplementary Material

Supplementary Table 1. Sensitivity analysis 1 (complete-case cohort): Baseline characteristics before and after 1:1 PSM with replacement.

| Variable | Pre-PSM | | | Post-PSM | | |
| --- | --- | --- | --- | --- | --- | --- |
|  | AZI (n = 167) | ERY (n = 135) | SMD | AZI (n = 133) | ERY (n = 133) | SMD |
| Male, n (%) | 88 (52.7%) | 66 (48.9%) | 0.076 | 70 (52.6%) | 65 (48.9%) | 0.075 |
| Age, years (median, IQR) | 4.00 (3.00, 7.00) | 6.00 (4.00, 9.00) | 0.492 | 7.00 (4.00, 9.00) | 6.00 (4.00, 9.00) | 0.212 |
| Severe phenotype, n (%) | 38 (22.8%) | 52 (38.5%) | 0.342 | 47 (35.3%) | 50 (37.6%) | 0.047 |
| Concomitant antibacterial therapy, n (%) | 141 (84.4%) | 123 (91.1%) | 0.204 | 113 (85.0%) | 121 (91.0%) | 0.185 |
| Concomitant antiviral therapy, n (%) | 113 (67.7%) | 94 (69.6%) | 0.042 | 81 (60.9%) | 92 (69.2%) | 0.173 |

PSM: propensity score matching; SMD: standardized mean difference; IQR: interquartile range.

Supplementary Table 2. Sensitivity Analysis 1 (Complete-Case Cohort): Three-Level Composite Clinical Efficacy Before and After 1:1 PSM (with Replacement)

| Outcome | Pre-PSM | | Post-PSM | |
| --- | --- | --- | --- | --- |
|  | AZI (n = 167) | ERY (n = 135) | AZI (n = 133) | ERY (n = 133) |
| Cure, n (%) | 84 (50.3%) | 62 (45.9%) | 65 (48.9%) | 62 (46.6%) |
| Improvement, n (%) | 75 (44.9%) | 67 (49.6%) | 54 (40.6%) | 65 (48.9%) |
| Ineffective, n (%) | 8 (4.8%) | 6 (4.4%) | 14 (10.5%) | 6 (4.5%) |
| Panel comparison | Unmatched ordinal test | | Paired matched ordinal test | |
| Ordinal test *P* value | Mann–Whitney U, *P* = 0.501 | | Wilcoxon signed-rank, *P* = 0.581 | |
| Distribution test *P* value | χ² test, P = 0.716 | | Bowker’s test, P = 0.236 | |

PSM: propensity score matching. Matching method: 1:1 nearest-neighbor matching with replacement using a caliper width defined as 0.2 × SD of the logit-transformed propensity score; the realized absolute caliper value in this sensitivity analysis was 0.175, restricting matched pairs to an absolute propensity score difference ≤ 0.175. Values are presented as n (%) or n. After matching, *P* values correspond to paired tests. Clinical efficacy was evaluated using a three-level composite ordinal endpoint.

Supplementary Table 3. Sensitivity analysis 2 (excluding double-missing cases): Baseline characteristics before and after 1:1 PSM with replacement.

| Variable | Pre-PSM | | | Post-PSM | | |
| --- | --- | --- | --- | --- | --- | --- |
|  | AZI (n = 577) | ERY (n = 302) | SMD | AZI (n = 286) | ERY (n = 286) | SMD |
| Male, n (%) | 297 (51.5%) | 150 (49.7%) | 0.036 | 142 (49.7%) | 147 (51.4%) | 0.035 |
| Age, years (median, IQR) | 4.00 (2.92, 6.00) | 5.00 (3.00, 8.00) | 0.32 | 5.00 (3.00, 7.00) | 5.00 (2.92, 7.00) | 0.054 |
| Severe phenotype, n (%) | 99 (17.2%) | 83 (27.5%) | 0.248 | 70 (24.5%) | 68 (23.8%) | -0.016 |
| Concomitant antibacterial therapy, n (%) | 499 (86.5%) | 265 (87.7%) | 0.038 | 247 (86.4%) | 250 (87.4%) | 0.031 |
| Concomitant antiviral therapy, n (%) | 386 (66.9%) | 220 (72.8%) | 0.13 | 197 (68.9%) | 205 (71.7%) | 0.061 |

PSM: propensity score matching; SMD: standardized mean difference; IQR: interquartile range.

Supplementary Table 4. Sensitivity Analysis 2 (Complete-Case Cohort): Three-Level Composite Clinical Efficacy Before and After 1:1 PSM (without Replacement)

| Outcome | Pre-PSM | | Post-PSM | |
| --- | --- | --- | --- | --- |
|  | AZI (n = 577) | ERY (n = 302) | AZI (n = 286) | ERY (n = 286) |
| Cure, n (%) | 255 (44.2%) | 122 (40.4%) | 119 (41.6%) | 117 (40.9%) |
| Improvement, n (%) | 314 (54.4%) | 170 (56.3%) | 159 (55.6%) | 160 (55.9%) |
| Ineffective, n (%) | 8 (1.4%) | 10 (3.3%) | 8 (2.8%) | 9 (3.1%) |
| Panel comparison | Unmatched ordinal test | | Paired matched ordinal test | |
| Ordinal test *P* value | Mann–Whitney U, *P* = 0.176 | | Wilcoxon signed-rank, *P* = 0.810 | |
| Distribution test *P* value | χ² test, P = 0.112 | | Bowker’s test, P = 0.319 | |

PSM: propensity score matching. Matching method: 1:1 nearest-neighbor matching without replacement with a caliper width of 0.086, restricting matched pairs to an absolute propensity score difference ≤ 0.086. Values are presented as n (%) or n. After matching, *P* values correspond to paired tests. Clinical efficacy was evaluated using a three-level composite ordinal endpoint.

Supplementary Table 5. Age-stratified LOS and treatment × age interaction test in the PSM-matched cohort

|  | AZI | ERY | Ratio (ERY/AZI) (95% CI) | *P* value |
| --- | --- | --- | --- | --- |
| <8 years | n = 285 | n = 287 | 0.99 (0.94, 1.04) | 0.666 |
| LOS, days, (median, IQR) | 6.90 (5.79, 8.82) | 6.95 (5.36, 8.06) |  |  |
| ≥8 years | n = 79 | n = 77 | 0.99 (0.90, 1.09) | 0.837 |
| LOS, days, (median, IQR) | 6.96 (5.73, 9.03) | 7.03 (5.98, 8.00) |  |  |
| *P*_interaction (treatment × age group) | | | >0.999 | |

LOS: length of stay; PSM: propensity score matching; CI: confidence interval.

Table S6. Age-stratified macrolide treatment duration and treatment × age interaction in the PSM-matched cohort

|  | AZI | ERY | Ratio (ERY/AZI) (95% CI) | *P* value |
| --- | --- | --- | --- | --- |
| <8 years | n = 285 | n = 287 | 1.44 (1.37, 1.52) | <0.001 |
| Macrolide treatment duration, days, (median, IQR) | 4.00 (4.00, 4.00) | 6.00 (5.00, 7.00) |  |  |
| ≥8 years | n = 79 | n = 77 | 1.60 (1.43, 1.79) | <0.001 |
| Macrolide treatment duration, days, (median, IQR) | 4.00 (4.00, 5.00) | 7.00 (5.00, 8.00) |  |  |
| *P*_interaction (treatment × age group) | | | 0.096 | |

PSM: propensity score matching; CI: confidence interval.

Supplementary Table 7. Age-stratified treatment escalation (systemic corticosteroid use) and treatment × age interaction in the PSM-matched cohort

|  | AZI | ERY | OR (95% CI) | *P* value | Paired McNemar *P* value |
| --- | --- | --- | --- | --- | --- |
| <8 years | n = 285 | n = 287 | 0.89 (0.66, 1.21) | 0.471 | 0.654 |
| Treatment escalation (systemic corticosteroid use), n (%) | 143 (50.2%) | 136 (47.4%) |  |  |  |
| ≥8 years | n = 79 | n = 77 | 0.95 (0.50, 1.82) | 0.885 | 0.761 |
| Treatment escalation (systemic corticosteroid use), n (%) | 43 (54.4%) | 41 (53.2%) |  |  |  |
| *P*_interaction (treatment × age group) | | | 0.858 | | |

PSM: propensity score matching; OR: odds ratio; CI: confidence interval. McNemar *P* values are reported as descriptive paired tests within each age stratum using exact binomial methods.

Supplementary Table 8. Safety Outcomes in the Full Cohort: Unadjusted (Fisher’s Exact Test) and Adjusted Analyses Using Firth Penalized Logistic Regression

| Safety endpoint | AZI (n=672) | ERY (n=377) | Unadjusted *P* value | Adjusted OR (95% CI) | Adjusted *P* value | Events |
| --- | --- | --- | --- | --- | --- | --- |
| Adverse events, n (%) | 2 (0.3%) | 7 (1.9%) | 0.013* | 6.52 (1.73, 24.53) | 0.006* | 9 |
| Hematologic event, n (%) | 1 (0.1%) | 0 (0.0%) | >0.999 | 0.67 (0.08, 5.42) | 0.703 | 1 |
| Gastrointestinal event, n (%) | 1 (0.1%) | 3 (0.8%) | 0.135 | 4.84 (0.95, 24.78) | 0.058 | 4 |
| Skin event, n (%) | 0 (0.0%) | 4 (1.1%) | 0.017* | 17.90 (1.56, 205.67) | 0.021* | 4 |

OR: odds ratio; CI: confidence interval. Unadjusted *P* values were computed using Fisher’s exact test. Adjusted ORs and 95% CIs were estimated using Firth penalized logistic regression to reduce small-sample bias and address outcome separation. Adjusted *P* values were derived from the Wald test of Firth penalized logistic regression coefficients (β).

Supplementary Table 9. Economic outcomes in the full unmatched cohort: macrolide drug costs and wastage

| Component | AZI | ERY | Unadjusted *P* | Adjusted effect | Adjusted *P* |
| --- | --- | --- | --- | --- | --- |
| Any wastage (>0 CNY) | 646/672 (96.1%) | 290/377 (76.9%) | <0.001 | OR 0.13 (0.09, 0.21) | <0.001 |
| Amount of wastage among patients with wastage (CNY) | n=646; median 53.35 (42.04, 59.82) | n=290; median 190.85 (119.28, 301.18) | <0.001 | Ratio 4.78 (4.42, 5.18) | <0.001 |

OR, odds ratio; CI, confidence interval; IQR, interquartile range. Unadjusted *P* values were computed using the Mann–Whitney test. Adjusted ORs for “Any wastage (>0 CNY)” were estimated using logistic regression with pair-clustered robust SEs. OR <1 favors ERY (lower odds of wastage). Cost ratios were obtained from Gamma-GLM with log link and exponentiated coefficients; ratio >1 indicates higher costs with ERY. Adjusted marginal means were derived by g-computation. Estimates should be interpreted cautiously due to sparse and skewed cost data.

Supplementary Table 10. Cohort-level macrolide costs: dispensed, actual expenditure, and avoidable wastage

| Group | Dispensed total (CNY) | Actual total (CNY) | Wastage total (CNY) | Wastage/dispensed, % |
| --- | --- | --- | --- | --- |
| AZI | 54,001.12 | 21,723.20 | 32,277.92 | 59.8% |
| ERY | 320,565.00 | 254,910.11 | 65,654.89 | 20.5% |
| Overall | 374,566.12 | 276,633.31 | 97,932.81 | 26.1% |
